# From paper to screen: regulatory and operational considerations for modernizing the informed consent process

**DOI:** 10.1017/cts.2022.379

**Published:** 2022-03-28

**Authors:** Nichelle L. Cobb, Dorothy F. Edwards, Erin M. Chin, James J. Lah, Felicia C. Goldstein, Cecilia M. Manzanares, Christine M. Suver

**Affiliations:** 1 Association for the Accreditation of Human Research Protection Programs, Inc. (AAHRPP), Washington, DC, USA; 2 University of Wisconsin-Madison School of Medicine and Public Health, Wisconsin Alzheimer’s Disease Research Center, Madison, WI, USA; 3 Emory University, Goizueta Alzheimer’s Disease Research Center, Atlanta, GA, USA; 4 Sage Bionetworks, Seattle, WA, USA

**Keywords:** Informed consent, eConsent, IRB, ethical oversight, regulation, Common Rule

## Abstract

Electronic platforms provide an opportunity to improve the informed consent (IC) process by permitting elements shown to increase research participant understanding and satisfaction, such as graphics, self-pacing, meaningful engagement, and access to additional information on demand. However, including these elements can pose operational and regulatory challenges for study teams and institutional review boards (IRBs) responsible for the ethical conduct and oversight of research. We examined the experience of two study teams at Alzheimer’s Disease Research Centers who chose to move from a paper-based IC process to an electronic informed consent (eIC) process to highlight some of these complexities and explore how IRBs and study teams can navigate them. Here, we identify the key regulations that should be considered when developing and using an eIC process as well as some of the operational considerations eIC presents related to IRB review and how they can be addressed.

## Introduction

Informed consent (IC) is a foundational component of ethical research. As articulated within the Belmont Report [1] and key US regulations governing human subjects research (i.e., the Common Rule [45 CFR 46.116]; Food and Drug Administration (FDA) regulations [21 CFR 50.20]), individuals must be given the opportunity, to the degree they are capable, to choose whether to participate in research. Legally effective IC is the process by which individuals (or their legally authorized representatives [LARs]) receive information about a research study in a language understandable to them, have sufficient opportunity to consider and discuss participation, and can decide whether to participate free from undue influence or coercion [2,3].

The need to make the IC process more meaningful and effective is well documented [4,5]. The process traditionally involves reading a lengthy and complex form that few people find helpful or comprehensible [6,7]. Challenges with IC prompted the US Department of Health and Human Services (HHS) to highlight the need for IC improvements in an Advanced Notice of Proposed Rulemaking, which noted the a) excessive length and technical language of IC documents and b) perception that IC forms tend to be more of a legal document meant to protect institutions rather than a decision-making resource for potential research participants [8]. This drive to improve IC resulted in new expectations within the revised 2018 Common Rule that explicitly requires the IC process to be organized and presented to facilitate participant comprehension.

Web-based technologies can enhance IC and provide an experience more similar to how most people access information in their everyday lives [9]. A growing number of electronic informed consent (eIC) platforms are available [10–12] that include some or all of the following:

- animation, videos, other visual imagery;
- avatars;
- questions to provide feedback on participant comprehension;
- popups and hyperlinks leading to supplemental information; and
- customized experience through the use of branching logic.

Incorporating features such as these in the eIC process provide attractive alternatives to address comprehension challenges, reach a wider range of participants, and engage those who might be excluded from research participation. For example, Sage Bionetworks pioneered a self-guided eIC tool that can be administered via a smartphone [13]. This is a convenient and effective way to engage more people and get them interested in participating in research because >85% of the US population owns a smartphone, with few differences seen across race, age, or socioeconomic status [14]. Further, the COVID-19 pandemic has underscored the need for research teams to conduct the IC process remotely to avoid in-person contact and potential exposure [15]. We expect that the trend toward eIC will continue post-pandemic.

This paper explores how IRBs and study teams can navigate applicable regulatory requirements when developing eIC processes and draws on the experience of Alzheimer’s Disease Research Center (ADRC) study teams who transitioned from a paper-based to an eIC approach that included a web-based tool. ADRC teams at Emory University and University of Wisconsin–Madison collaborated with Sage Bionetworks (together “we” or the “team”) to reimagine the IC process for nontherapeutic studies that recruit a) individuals with potential memory disorders and decreased decision-making capacity and b) healthy controls. We produced a participant-centric, interactive eIC experience that incorporates IC best practices such as using simplified language, graphics, comprehension questions with corrective feedback [16–18], and tiered information (i.e., allowing participants to access supplemental information about specific concepts). This eIC approach addressed some of the barriers to an effective IC process that ADRC study coordinators previously identified, including making it easier to customize the order in which information is presented to research participants [19]. The eIC experience was piloted with study coordinators and participants who provided input on which features of eIC were most relevant and meaningful to them (manuscript in preparation). Although our eIC development considered the needs of individuals with potential memory deficit and some cognitive impairments, we think the approaches we adopted are broadly applicable.

### eIC Regulations Considerations

Presenting information to potential participants or their LARs (henceforth collectively ‘“participants”) in an electronic format meets the definition of eIC in the 2016 Office for Human Research Protections (OHRP) and FDA joint guidance (henceforth “OHRP/FDA guidance”) [20], which outline an expansive view of eIC, stating it encompasses: “the use of electronic systems and processes that may employ multiple electronic media, including text, graphics, audio, video, podcasts, passive and interactive web sites, biological recognition devices, and card readers, to convey information related to the study and to obtain and document informed consent.”

This definition emphasizes that eIC is more than obtaining an electronic signature: the learning experience is an integral part of the IC process. As part of our development process, it was crucial to identify which regulations applied to the ADRC studies to ensure compliance and because of important differences between the major US federal regulations that apply to human research. For example, the Common Rule and FDA regulations governing human subjects research differ regarding the requirements for electronic signature. The Common Rule simply communicates that consent forms may be signed electronically (45 CFR 46.117)]. In comparison, FDA regulations (21 CFR 11 aka “Part 11”) include detailed requirements that must be met to ensure electronic signatures are “trustworthy, reliable, and generally equivalent to a handwritten signature executed on paper” [21]. Part 11 includes requirements for any system capturing an electronic signature to be secure with restricted access, include a method for validation, and produce an audit trail. The ADRC studies for which the eIC tool was developed were not subject to FDA regulations and thus were not required to comply with Part 11. Nonetheless, we highlight areas where compliance with FDA regulations differs from the Common Rule and those we determined applied to the ADRC studies (Table 1).

**Table 1.** Key regulations that should be considered when developing an electronic informed consent (eIC) process

Regulation	Relevance	Applied to ADRC Study
Common Rule	• Informed consent (IC) must be documented by means of a “written” IC form (unless this requirement is waived by an institutional review board [IRB]), which encompasses electronic format • Outlines the requirements for legally effective IC, which include: ○ documentation of IC ○ the circumstances under which IC can be sought ○ presenting information in a language understandable to participants ○ providing participants with information that a reasonable person would want to have to make an informed decision about whether to participate and an opportunity to discuss that information ○ organization of the information provided to participants • Identifies when an IRB can waive or alter IC or waive documentation of IC • Stipulates certain aspects of IC content, including: ○ a requirement to begin with a concise and focused presentation of the key information ○ basic and additional elements of IC • Describes requirements for posting clinical trial IC forms NOTE: Subparts B and D identify additional requirements regarding from whom assent, IC or permission must be obtained	Yes
FDA	*21 CFR 50* • Similar to the Common Rule but does not specify IC organization, does not require a concise and focused summary, and does not define what “written” format encompasses • Permits limited exceptions to the requirement to obtain IC *21 CFR 11* • Addresses requirements for the use of electronic systems to maintain and generate records, such as expectations for system validation, controls, workflow, standard operating procedures, audit trails, and data backup • Identifies requirements for electronic signatures, including uniqueness and identity verification	No
HIPAA Privacy and Security Rules	• Do not address nor preclude electronic signatures to use and disclose protected health information • Electronic signatures must be compliant the Federal Electronic Signatures in Global and National Commerce (ESIGN) Act and the Uniform Electronic Transactions Act (UETA)	Yes

ARDC = Alzheimer’s Disease Research Center.

Another regulation to consider is whether the HIPAA Privacy Rule applies and, if so, whether a separate authorization to use protected health information will be required. Additional examples of regulations that may apply to some research and affect the eIC process include the Children’s Online Privacy Protection Act (COPPA) [22] and Family Education Rights and Privacy Act (FERPA) [23], which may influence from whom permission must be obtained and documented.

### eIC Tool Features

This project presented the team the opportunity not only to reformat the current paper IC version into an eIC but also to add key features to create a participant-centered IC experience by:

- Simplifying the language and presenting information in modules.
- Representing themes with iconography.
- Allowing participants to choose the order they view the modules.
- Providing optional (tiered) information about specific concepts.
- Concluding each module with questions to reinforce learning and allow self-assessment of comprehension of the material.

**Fig. 1.**
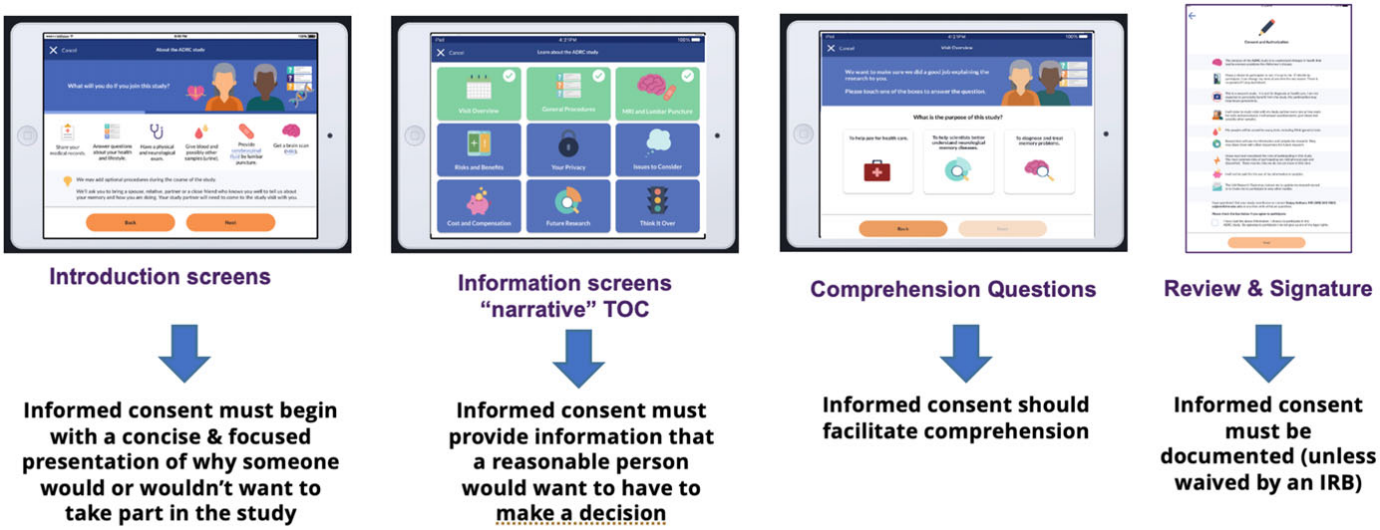
Electronic informed consent flow and features. IRB, institutional review board.

The eIC we developed employed best practices for the presentation of information in an online format as implemented in Apple ResearchKit, the National Institutes of Health *All of Us* Research Program [24], and the HHS and the US General Services Administration’s Research-Based Web Design & Usability Guidelines [25]. Figure 1 illustrates the navigation through our eIC.

In accordance with the revised Common Rule, our eIC tool starts with a concise summary of the study, followed by study details grouped into a table of contents presented in a 3 × 3 grid. Each “tile” represents a module and includes a title and icon that conveys the section’s content. The modules represent essential sections of an IC, such as risks and benefits; privacy; costs and compensation. The reader must click on all modules, moving through the content and answering questions to assess understanding before being allowed to access the signature section. In each module, optional information is available about key concepts via hyperlinks in popup windows. After reviewing each module’s information and answering the comprehension questions, a summary of main concepts reinforced by the iconography used throughout the eIC is shown so readers can verify their understanding and consolidate their decision on whether to join the study.

Because ADRC researchers enroll individuals with memory deficits, an important feature of the eIC tool is the addition of multiple-choice questions after each module rather than at the end of the IC interaction; this deliberate design feature assesses comprehension of each module’s concepts rather than overall recall. It is a well-accepted approach in AD research used to address comprehension of individual elements in competency assessment [26] and can function as a “cognitive prosthetic” to help potential research participants, especially those with mild cognitive impairments, stay focused on the IC session [27]. When participants select a response, the tool provides feedback on whether the response was correct and, if not, what the correct response was and why. Questions were designed to be engaging and reinforce learning. Asking questions about specific study concepts and providing corrective feedback is an effective way to determine if participants comprehend the information [28]. Answers can help identify misunderstandings and lead to conversations to address knowledge gaps, which is especially important when working with individuals with potential memory and cognitive deficits, but also a best practice for IC generally.

### eIC Regulatory Oversight and Logistical Challenges

Federal regulations do not directly describe the specific information an IRB must obtain to review an eIC process. One reason for this lack of specificity is that IRBs must make their determinations independent of format when they review the IC process and any information provided to participants. A key IRB responsibility is to ensure participants receive any information, including the basic elements of consent and any relevant additional details, in “understandable language.” The 2018 Common Rule obligates IRBs to ensure the IC process facilitates comprehension and includes information that a reasonable person would want to have to make an informed decision about whether to participate. Additionally, federal regulations require IRBs to maintain documentation of their reviews, including any study changes or IC revisions.

During the development of the eIC tool, ADRC researchers, in conjunction with their local IRBs, worked through regulatory and operational issues that the review of an eIC process presented. Not all IRBs have the bandwidth for such close collaboration. Consequently, Table 2 provides practical recommendations for IRBs and research teams considering eIC with interactive features, focusing on four areas:

1. Presenting the eIC experience to the IRB and tracking changes.
2. Retaining records
3. Documenting the IC
4. Providing a copy of the IC

**Table 2.** Key considerations and recommended practices for electronic informed consent (eIC) approaches

Operational Considerations	Regulatory Considerations	Recommended Practices for Research Teams and Institutional Reviewer Boards (IRBs)
Presenting an eIC experience to the IRB	IRBs must be able to assess all aspects of an informed consent (IC) process, including any dynamic or interactive features, and their review should include: • All informational materials including written materials, videos, and web-based presentations, an individual will receive or view during the eIC process. • Any optional questions asked or methods used to gauge the comprehension of key study elements and the usability of eIC materials to ensure they are easy to navigate. • Hyperlinked content to ensure study-related information there is accurate and appropriate.	Suggested approaches for assessing interactive features include having research teams provide: • Hyperlinks to the draft eIC materials so the IRB can experience the interactive features as participants, or • Screenshots of each eIC screen with annotations describing the interactive features and actions that can be taken from the page (e.g., clicking on a hyperlink will define highlighted terms or show details about a study procedure) and indicate allowed navigation between screens, or • A video demo that records the IC experience/content, which can be uploaded and archived within an electronic IRB system. This last approach requires the study team to create the video, which can be time-consuming and require specific expertise.
Tracking changes to the IC	If a copy of the IC provided to participants includes one or more hyperlinks to information, then the hyperlinks should be maintained and information accessible to participants until the study is complete.	To track revisions requested by the IRB or future updates, IRBs might consider having research teams create and maintain one or more of the following: • A text document to accompany any visual presentation and within which changes in IC content and presentation can be tracked. Any websites or information accessed within the IC by hyperlink should be captured as well, such as by creating PDF versions or screenshots of them. • A log of changes to the IC that captures both content and format changes. This log would identify the version date of the IC, when the IRB approved it, and the changes incorporated into that version. • Webpages that capture the content and features of each version of the IC approved by the IRB. Several solutions exist to archive public and nonpublic websites.
Providing participants with a copy of the IC	Federal regulations require IC to be documented via a written, IRB-approved IC form unless the IRB waives the requirement either to obtain IC or documentation of IC. The Common Rule a) defines written as including information provided in electronic format (45 CFR 46.102(m)) and b) requires a “written copy” be given to the person signing the IC document (45 CFR 46.117(a)). OHRP/FDA guidance states that if the eIC uses hyperlinks or other websites or podcasts to convey information specifically related to the research, then the information in the hyperlinks should be included in any printed paper copy provided.	Research teams can provide participants with a summary that highlights key information about the research study and, which participants can either print either in tangible form or save electronically for easy reference. The summary should include a link to all IC materials.
Retaining records	All IRB-approved eIC versions, including copies of each eIC version signed by individual participants, must be maintained in compliance with applicable regulations; federal regulations require IRBs to maintain documentation for at least 3 years after a study’s closure. Sponsor or institutional requirements might require researchers to retain documents for longer.	IRBs should ensure a document retention plan is in place, both for their and research team records related to eIC. The research team should provide its standard operating procedure or describe its record retention as part of the IRB application. Procedures should consider a backup plan in case hyperlinks within a document or linked information cease to exist, to ensure a participant has ongoing access to it or an auditor can see the same information the IRB and participants viewed.
Documentation of informed consent, including electronic signature	If an IRB requires documentation of informed consent and the research is FDA-regulated research, the electronic system must comply with Part 11 requirements and capture and record the date that the participant or participant’s LAR provides consent. Both the FDA and the Common Rule regulations permit IRBs to waive documentation of IC when the research involves no more than minimal risk and involves no procedures for which written consent is normally required outside of the research context.	IRBs can assist study teams by helping them identify the relevant regulations that apply to their studies and providing flexibility regarding when and how written IC is obtained, as appropriate. For example, IRBs should identify for research teams which eIC systems they can use that are Part 11 compliant for research that is FDA-regulated as well as acceptable methods for capturing an electronic signature, based on FDA guidance, including Conduct of Clinical Trials of Medical Products During the COVID-19 Public Health Emergency: Guidance for Industry, Investigators, and Institutional Review Boards (https://www.fda.gov/regulatory-information/search-fda-guidance-documents/fda-guidance-conduct-clinical-trials-medical-products-during-covid-19-public-health-emergency). If a study is not FDA-regulated, IRBs should not require research teams to use Part 11 compliant systems and permit a broader range of signature options (e.g., REDCap that is not Part 11 compliant), so that alternate approaches to eIC can be explored that allow more flexibility in terms of how an electronic signature can be captured. In the case of minimal risk research, IRBs should consider waivers of documentation of IC (as well as waivers of HIPAA authorization), when possible, regardless of whether the research is FDA-regulated.

IRBs and study teams should work together to develop a plan that addresses each of these areas. For example, research teams should discuss with the IRB what constitutes the “copy” of the IC when it includes interactive elements and how the copy should be provided (e.g., on an electronic storage device, via email, or another method). We recommend research teams provide participants with a summary that highlights key information about the research study and which participants can either print in tangible form or save electronically for easy reference. This approach mirrors the concise and focused summary requirement in the Common Rule (45 CFR 46.116(a)(5)) and is similar to the short form approach often used to document IC when the contents of the IC are presented orally to a participant. When using the short form, the study team must create and provide a written summary of what is to be said to the prospective participant and include an acknowledgment that all applicable elements of IC were presented. For an interactive eIC experience, this “written summary” of key information with the link to the eIC process could be given to participants in paper format for the participant record.

One consideration not driven by federal regulations is the common practice of IC form stamping. IRBs often add approval stamps on IC forms to assist study teams with tracking approved document versions. Stamps frequently identify the IRB, IC version, approval date, and study expiration date. Some platforms (e.g., REDCap) can generate a static capture of IC document screens and convert them to PDF files. Many IRB systems can stamp PDFs. IRBs and study teams need to be flexible and develop procedures, such as the REDCap alternative method, to identifying IRB-approved documents. The eIC approach with interactive features that our team developed, for example, cannot be stamped in the same way as traditional IC forms, but text within our online forms could show the version and IRB approval date, essentially recreating the information in an IRB stamp. Additionally, study teams can describe in standard operating procedures their process for a) tracking changes and versions of interactive eIC forms and b) presenting the correct versions to participants. If IRBs do not require written representation of IC information to be stamped, this challenge is completely avoided.

### Core Versus Optional Information

The revised Common Rule placed IC front and center, reemphasizing the need for study teams and IRBs to carefully consider IC content and presentation. An interactive eIC tool can better meet some of the expectations outlined in the Common Rule for IC compared with a paper-based or eIC approach alone because the interactive features give participants more agency in determining the level of detail and order in which to consume the information. An interactive eIC is responsive to two key requirements in the Common Rule:

1. Prospective participants must be given information that a reasonable person would want to evaluate to make an informed decision about participating and an opportunity to discuss that information.
2. The information given should be in a language understandable to participants.

Although being able to tailor the level of detail and complexity of information is not unique to an eIC approach, eIC facilitates it through hyperlinks and pop-ups.

The Common Rule uses the reasonable person standard to guide IC content, but does not define *reasonable person*. Operationalizing this standard is challenging because individuals differ in what information they want about research [29]. The challenge of defining what a reasonable person would want to know is compounded by the fact that participants differ in the information they consider to be important about research studies. For example, one study showed most potential participants wanted to know information about the return of research results (91%) [30], yet federal regulations do not prioritize disclosure of clinically relevant results either as a concept that should be included in the concise and focused summary or even as a basic element of IC. Additionally, the Common Rule requires that for a reasonable person to make a decision, an IC must be presented in language understandable to them—but ensuring all IC language is understandable to every possible participant is a difficult standard to meet [31]. As the FDA notes, “understandable” means the information is presented to potential participants “in a language and at a level the participants can comprehend (including an explanation of scientific and medical terms)” [32].

The ADRC eIC experience was responsive to the reasonable person standard and providing information in language understandable in two key ways. Specifically, the ADRC eIC incorporated:

1. A tiered-information approach that ensured individuals received core information about the research and allowed them to access supplemental information as desired.
2. A modular approach, iconography, simplified language (middle-school reading level), definitions, and glossary of terms such that individuals of varied educational levels could learn about the study.

Further, grouping information into logical modules that met the Common Rule requirement for IC organization presentation.

**Fig. 2.**
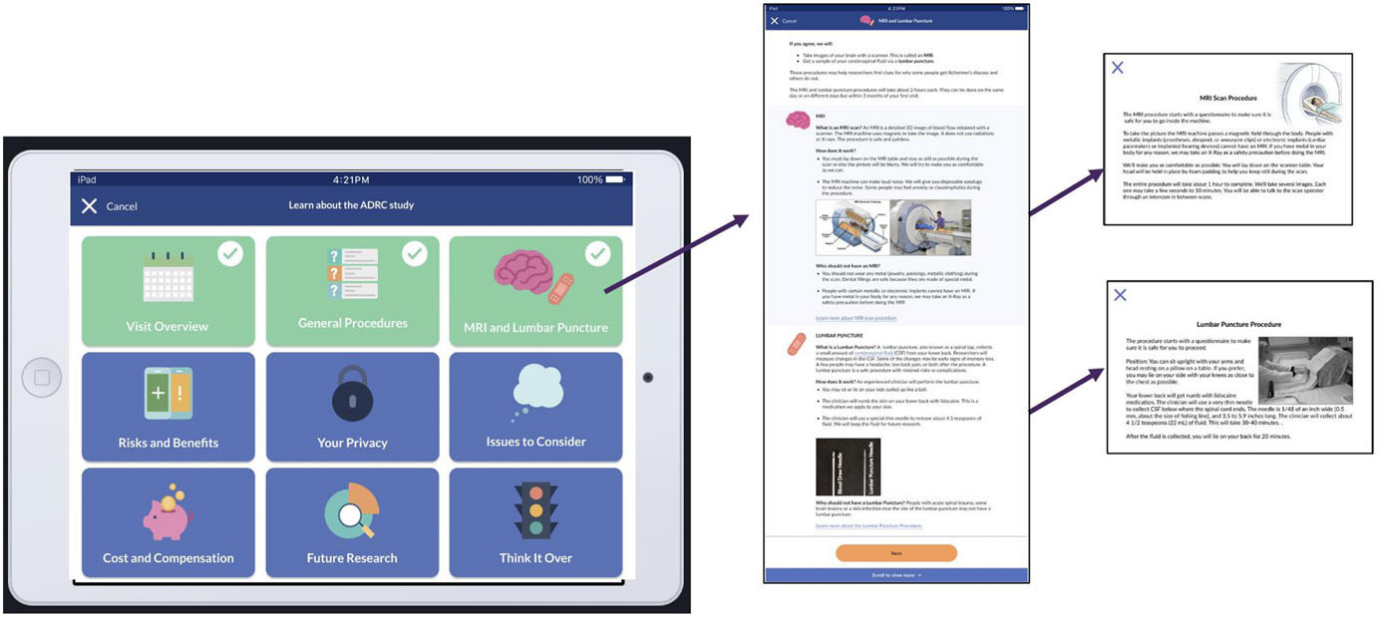
Electronic informed consent tiered information feature

A decision our team made in collaboration with the local IRBs was determining what information could be tiered, resulting in some information being presented to all (i.e., core information), while supplementary information would be accessible on-demand via a pop-up window if the reader clicked the hyperlink (i.e., optional information). Core information included required basic and applicable additional elements of IC per the Common Rule. A simple example of the tiered approach appears in the IC section that describes study visits: “We will ask you to return for follow-up visits every one or two years for as long as you can.” This statement meets the basic element of IC to inform individuals of the expected duration of their study participation. One could click on the phrase “as long as you can” to read details about long-term participation, and a pop-up window appeared that provided details (i.e., the possibility that if participants can no longer attend visits in person, they may be able to participate via telephone and their information would be collected from a surrogate if they became too ill to continue via telephone). The IRB required the estimation of study duration (“as long as you can”) to appear within the body of the IC materials as a required basic element of IC, while the detail about how participation might change from in-person to remote visits was viewed as of interest to some individuals but not essential to decision-making. For the tiered approach, each module starts with core information and includes links to pop-up screens for optional information (Figure 2); this approach allows individuals to tailor the IC process to the level of information they need and how they prefer to access it.

### Other Considerations

Several additional factors can affect the development of eIC, such as:

- **State laws**: Some states have restrictions on what platforms may be used to capture electronic signatures, which can limit what platforms or approaches can be considered for eIC, or dictate specific requirements for IC language and font size (e.g., California’s Protection of Human Subjects in Medical Experimentation Act that requires the “Experimental Participants Bill of Rights” be provided to all participants in medical research and be in a specific format).
- **Single IRB**: As of January 20, 2020, most federally supported, multi-site research must be overseen by a single IRB (45 CFR 46.114(b)). If a single IRB will be used for the study, any local context requirements related to eIC will need to be communicated to the reviewing IRB [33]. The process for including site-specific language within an eIC should be considered and discussed with the single IRB.
- **Posting Clinical Trial Consent Forms:** The revised Common Rule includes a new provision that a copy of the final version of the consent form is posted on a publicly available federal website for each clinical trial conducted or supported by a Common Rule department or agency (45 CFR 56.116(h)).
- **Non-English Speakers**: The Common Rule requirement for research teams to provide information to participants in language understandable to them does not just speak to readability but also whether what language a participant may comprehend. Translation and interpretation needs should be factored into the development of any IC process, whether paper- or electronic-based.
- **Assent**: For studies involving children that use an eIC process, the research team will need to consider the development of age-appropriate materials if an assent process is required. Use of any IC materials aimed at parents or guardians may only be suitable for older children.

Research teams developing eIC processes should consult with their IRB early on to ensure the team addresses applicable regulations, understands IRB review requirements, and factors in tracking, versioning, and maintaining materials involved in an eIC process. To facilitate collaboration, we developed questions to help research teams and IRBs anticipate and address regulatory and other issues that innovative eIC processes can present (Table 3).

**Table 3.** Key operational and regulatory questions to ask when developing an electronic informed consent (eIC) approach

Operational
• What materials related to the eIC does an institutional reviewer boards (IRB) need to review? • How should any applicable interactive elements be presented to the IRB? • How should changes to the informed consent (IC) be tracked? • How should the versions of the eIC be tracked? • What constitutes the copy of the IC that should be provided to participants? • What alternatives should be offered for participants who do not have access to the internet or are not comfortable with eIC? • If a single IRB will review the research study, how should site-specific information be incorporated into the eIC?
Regulatory
• Which regulations apply to the study? • Is documentation of informed consent required? • Are there state or local laws or organizational policies that could affect an IC process? • If the study is a clinical trial, how will the research team represent their eIC on a publicly available Federal website?

## Conclusion

Interviews with study coordinators from the two ADRC teams about their paper-based IC process identified key challenges: difficulties maintaining participant attention, explaining complex procedures, and modifying the IC process to support the needs of individual participants [19]. Often, such challenges are not specifically considered as part of IRB review. IRBs traditionally have focused more on forms than processes, but the process can be as or more important in promoting robust IC [34]. Moreover, IRBs must ensure that eIC approaches do not simply reproduce the content of an IC form in an electronic format but instead improve the IC process [9]. An interactive eIC experience inherently shifts the focus from reading a form to the learning *process* and should prompt research teams to describe in more detail, and IRBs to consider in more depth, how participants engage in the IC process and access information, how information is organized and presented, and how understanding is gauged. Thinking more deeply about the IC process could lead to wider adoption of interactive eIC experiences that enhance participant engagement and understanding and reduce potential selection bias [9].

Use of eIC, including more platforms with interactive features, was increasing but accelerated during the COVID-19 pandemic [11,15], highlighting how well IRBs and study teams can work together to facilitate the transition from paper-based, in-person IC to a remote eIC process. Increased use of eIC likely will become a mainstay of research and provides an opportunity to better meet the expectations for IC outlined in the revised Common Rule, thus substantively improving participant experience by leveraging interactive features that enable the IC process to be tailored to individual participants’ needs.
